# Platelet activation and aggregation by the opportunistic pathogen *Cutibacterium (Propionibacterium) acnes*

**DOI:** 10.1371/journal.pone.0192051

**Published:** 2018-01-31

**Authors:** Frida Petersson, Ola Kilsgård, Oonagh Shannon, Rolf Lood

**Affiliations:** 1 Division of Infection Medicine, Department of Clinical Sciences Lund, Lund University, Lund, Sweden; 2 Department of Immunotechnology, Faculty of Engineering Lund, Lund University, Lund, Sweden; Royal College of Surgeons in Ireland, IRELAND

## Abstract

*Cutibacterium* (*Propionibacterium*) *acnes*, considered a part of the skin microbiota, is one of the most commonly isolated anaerobic bacteria from medical implants in contact with plasma. However, the precise interaction of *C*. *acnes* with blood cells and plasma proteins has not been fully elucidated. Herein, we have investigated the molecular interaction of *C*. *acnes* with platelets and plasma proteins. We report that the ability of *C*. *acnes* to aggregate platelets is dependent on phylotype, with a significantly lower ability amongst type IB isolates, and the interaction of specific donor-dependent plasma proteins (or concentrations thereof) with *C*. *acnes*. Pretreatment of *C*. *acnes* with plasma reduces the lag time before aggregation demonstrating that pre-deposition of plasma proteins on *C*. *acnes* is an important step in platelet aggregation. Using mass spectrometry we identified several plasma proteins deposited on *C*. *acnes*, including IgG, fibrinogen and complement factors. Inhibition of IgG, fibrinogen or complement decreased *C*. *acnes*-mediated platelet aggregation, demonstrating the importance of these plasma proteins for aggregation. The interaction of *C*. *acnes* and platelets was visualized using fluorescence microscopy, verifying the presence of IgG and fibrinogen as components of the aggregates, and co-localization of *C*. *acnes* and platelets in the aggregates. Here, we have demonstrated the ability of *C*. *acnes* to activate and aggregate platelets in a bacterium and donor-specific fashion, as well as added mechanistic insights into this interaction.

## Introduction

*Propionibacterium acnes* is a predominant skin bacterium that primarily colonizes sebum rich areas, and is suggested to benefit our health [1]. Due to its presence on skin, and its genetic diversity from several other *Propionibacteria*, it has been proposed to rename *P*. *acnes* to *Cutibacterium acnes* [2] and divide the different types into *C*. *acnes* subsp. *acnes* (type I), *C*. *acnes* subsp. *defendens* (type II), and *C*. *acnes* subsp. *elongatum* (type III) [3]. *C*. *acnes* is associated with several diseases, including acne [4], infective endocarditis (IE) [5] and localized chronic inflammation on medical implants due to biofilm formation [6]. By further genetic analysis and subdivision of *C*. *acnes* into sequence types by multi-locus and single-locus sequence typing, it has been demonstrated that specific types of *C*. *acnes* are associated with disease, while others are associated with health [4,7–11]. Several emerging pathogens have earlier been considered bacteria with low virulence, only being able to cause damage during specific circumstances. The novel role of these bacteria is partly driven by advances in the medical field, including the now common use of joint prostheses and catheters; commonly being contaminated by the skin microbiota, including *Staphylococcus epidermidis* and *C*. *acnes* [12], showing a localization-triggered bacterial pathogenesis [13].

Though not the main microorganism identified from infective endocarditis, there is an increasing number of reports of *C*. *acnes* associated with this pathogenesis [14]. The impact of this should not be neglected since *C*. *acnes* is highly immunostimulatory [15]. Due to its strong Th1 mediated response, *C*. *acnes* has historically been used experimentally to trigger immune responses for tumor treatment in rodents [16]. During these experiments, it became evident that *C*. *acnes* had an adverse effect on circulating immune cells, generating thrombocytopenia and decreased fibrinogen levels in mice [17,18], indicating an interaction between *C*. *acnes* and the coagulation system, including platelets. However, no mechanism(s) to describe the thrombocytopenia or effect on the coagulation system has been given.

Platelets play a critical part in coagulation and immune response [19]. These small fragmented progeny of megakaryocytes have recently been shown to interact with many bacteria, such as *Streptococcus pyogenes* [20], *Staphylococcus aureus* [21], *Enterococcus faecalis* [22], and *Aerococcus urinae* [23]. Often, this interaction results in platelet aggregation and is dependent on direct (bacteria-platelet) or indirect (bacteria-plasma component-platelet) interactions, with the latter usually being mediated by fibrinogen, complement, and IgG [24]. This is reflected in a short lag-time before platelet aggregation for direct interactions and a long lag-time for indirect interactions that are dependent on the accumulation of plasma proteins to the bacterial surface before aggregation can occur [24]. In addition, the interaction between platelets and bacteria can result in platelet activation. However, this is often dependent on a secondary co-signal, usually specific IgG against the bacteria that interact with platelet Fc-receptors [24–28]. In turn, this can lead to thrombus formation [29], and release of cytokines and antimicrobial peptides [30]. On the one hand, this aids the clearing of the pathogen by recruitment of other immune cells [31], and by entrapment in a thrombus containing antibacterial substances [32]. On the other hand, this may contribute to the pathogenesis of many infections, such as IE and septicemia [33,34].

In light of the demonstration that *C*. *acnes* forms biofilms on medical implants and catheters in plasma rich environments, induce thrombocytopenia in mice, and being indicated as one of the foremost contaminants in platelet concentrates [35–37], we believe it to be of importance to elucidate the interaction of *C*. *acnes* and platelets, and the downstream effects of such an interaction.

## Materials and methods

### Bacterial strains and growth conditions

The isolated *C*. *acnes* strains are described elsewhere [6,38] and can be found in S1 Table. *C*. *acnes* was routinely grown in Tryptic Soy Broth (TSB; Bacto BD, Sparks, MD, USA) supplemented with 1.5% glycerol, under anaerobic conditions (10% H_2_, 10% CO_2_, 80% N_2_) at 37°C.

### Blood collection and preparation

Blood samples from healthy donors were collected with 0.1 M Na_3_-citrate as an anticoagulant. Platelet-rich plasma (PRP) and platelet-poor plasma (PPP) was isolated through centrifugation (150 g 15 min; 2000 g 10 min, respectively). To obtain serum, blood was collected in empty tubes, and allowed to coagulate. Cells or blood clots were pelleted through centrifugation (1500 g, 10 min), and the supernatant saved. Sampling of blood from healthy volunteers giving informed consent has been approved by the regional Ethical Review Board in Lund, Sweden (approval 2008/657), and in accordance with the Declaration of Helsinki.

### Platelet aggregation

The platelet aggregation response to bacteria was investigated using a platelet aggregometer (ChronoLog 490, Havertown, PA, USA). PRP (450 μl) was added to pre-heated glass tubes placed in sample wells, and 50 μl bacteria suspension (1x10^8^ cfu/ml) were added while stirring. For dose-dependency experiments, bacteria at a concentration of 1x10^8^ cfu/ml were serial diluted in PBS to generate two-fold dilutions while maintaining the same volume (e.g. 6x10^6^-1x10^8^ cfu/mL). PPP was used as a reference, and collagen I (5 μg/ml; ChronoLog Corp.) as a positive control. The software AggroLink was used for all analyses. For certain experiments, pre-treatment of PRP with IdeS (20 μg/ml, 30 min RT), anti-FcRγIIA (AT10, 100 μg/ml, 15 min 37°C)(Serotec, Oxford, UK), or prostaglandin E (PGE_1_, 1 μM; Sigma-Aldrich, St. Louis, MO, USA), was conducted. IdeS is a specific IgG protease with no other known activities in plasma or elsewhere [39], hydrolyzing IgG in the hinge region, inhibiting the downstream effects of IgG signaling [40,41]. AT10 is a specific FcRγIIA antagonist, thus blocking all signaling through this receptor. For pre-absorption of plasma proteins to *C*. *acnes*, bacteria (50 μl; final concentration 1x10^8^ cfu/ml) was incubated with PPP, serum or heat inactivated serum (100 μl, 15 min 37°C) or PBS (control) and washed in PBS before being added to PRP.

### Platelet activation and flow cytometry

Flow cytometry was used to investigate the platelet activation in response to *C*. *acnes*. PRP (20 μl) was diluted in 30 μl HEPES buffer pH 7.4 and incubated with 5 μl bacteria (1x10^8^ cfu/ml, final concentration) at room temperature for 25 minutes. ADP (5 μM, Sigma-Aldrich, St. Louis, MO, USA) or thrombin (1 U/ml, ChronoLog, Havertown, PA, USA) were used as positive controls for platelet activation. Fluorescent antibodies (1:10, 15 min at room temperature, dark) were added to detect the platelet population (CD42a PerCP, BD, Sparks, MD, USA), surface-bound CD62P (CD62P PE (BD, Sparks, MD, USA)), and platelet activation through presentation of an activation dependent epitope on GPIIb/IIIa (PAC-1 FITC, BD, Sparks, MD, USA). To determine the role of IgG and fibrinogen in platelet activation by *C*. *acnes*, PRP was pre-treated for 15 minutes at room temperature with AT10 (100 μg/ml) and ReoPro (Abciximab, 10 μg/ml, Centocor, New York, NY, USA) prior to addition of bacteria and platelet antibodies. All samples were acquired and analyzed using a BD Accuri C6. The results are presented as median fluorescence intensity (MFI) fold increase in relation to the signal in the control sample with PRP and HEPES buffer alone for each experiment. Experiments examining platelet activation were not necessarily conducted concurrently.

### Platelet protection of *C*. *acnes*

Bacteria (50 μl; 1x10^8^ cfu/ml final concentration) were incubated alone or with 450 μl PRP or PBS at 37°C while shaking (700 rpm) for 0, 25, and 120 minutes. The experiment was performed with and without the neutrophil antimicrobial peptide LL37 (100 μg/ml), as a control for bacterial killing. The mixture was either vortexed or sonicated (0.5 cycles, 90 amplitude, 10 x 10 seconds with 10 seconds rest between each sonication) as described recently [20] before being plated on TSBA plates. Plates were incubated anaerobically until colonies were visible, and cfu/ml could be determined.

### Determination of anti*-C*. *acnes* titers in plasma

Bacteria (5 μl, 1x10^8^ cfu/ml) were incubated with 50 μl plasma from different healthy donors for 15 minutes at room temperature. Bacteria were washed with PBS and incubated with FITC-labeled mouse anti-human IgG (1:50) (15 minutes, room temperature, dark). Bacteria pre-incubated in PBS (instead of plasma) were used to control for non-specific binding. Before analysis using flow cytometry, the samples were washed and resuspended in PBS.

### Shotgun mass spectrometry analysis of plasma-adherent proteins

*C*. *acnes* strains KPA171202 and AS12 were grown anaerobically in TSB supplemented with 2% plasma until reaching stationary phase. Pelleted and washed bacteria were lysed with a Bead-beater (12x30 sec, 1600 rpm), and the resulting supernatant dried. The samples were alkylated and reduced, before treatment with trypsin overnight at 37°C. The samples were analyzed on a Q Exactive Plus mass spectrometer (Thermo Scientific, Grand Island, NY, USA) connected to an EASY-nLC 1000 ultra-high pressure liquid chromatography system (Thermo Scientific, Grand Island, NY, USA). Peptides were loaded on an EASY-Spray column (Thermo Scientific; ID 75 μm x 25 cm) at a constant pressure of 600 bars, and separated at a flow rate of 300 nl/min. Peptides were eluted with a linear gradient from 95–35% solvent A (0.1% formic acid in water) and 5–65% solvent B (0.1% formic acid in acetonitrile) over 60 min. The precursor ions were fragmented using high-energy collision induced dissociation (HCD) at normalized collision energy of 30. Acquired spectra were searched with X!Tandem [42] against the Human reference proteome (UP000005640), extended with reverse decoy sequences for all entries. Obtained peptide spectrum matches were statistically evaluated using PeptideProphet and protein inference by ProteinProphet, both part of the Trans Proteomic Pipeline (TPP, v4.7 Polar Vortex)[43]. A minimum protein probability of 0.9 was set to match a false discovery rate (FDR) of <1%. The resulting pep.xml and prot.xml files were used as input for the spectral counting software tool MAYU [44].

### Microscopy

Fluorescence microscopy was used to visualize the *C*. *acnes*-induced platelet aggregates. *C*. *acnes* was added to PRP as previously described. For *C*. *acnes* labeling DAPI and Oregon Green were used as indicated. PBS, PPP and PRP alone were used as negative controls, and collagen I as a positive control for platelet aggregation. The samples were fixed in 4% paraformaldehyde for 45 minutes and washed in D-PBS supplemented with 5% BSA and 50 mM glycine. Primary antibodies were added (mouse anti-human CD61 1:500, BD, Sparks, MD, USA) and incubated for 1 hour at room temperature, before washing in PBS + 5% BSA, and addition of secondary and/or labeled primary antibodies (Alexa Fluor 594 labeled anti-mouse IgG 1:500, Invitrogen, Grand Island, NY, USA; Alexa Fluor 647 labeled goat anti-human IgG 1:1000, Jackson ImmunoResearch, West Grove, PA, USA; FITC labeled rabbit anti-human fibrinogen 1:500, Dako, Santa Clara, CA, USA), continuing incubation for 1 hour. Finally, the samples were washed and re-suspended in PBS + 5% BSA and adsorbed on pre-coated poly-L-lysine coverslips for one hour before mounting to slides using DAPI ProLong Gold mounting medium (Invitrogen, Grand Island, NY, USA). The samples were allowed to settle overnight and analyzed in a Nikon Eclipse T*i* microscope.

### Statistical analysis

Prism 7 (GraphPad software, La Jolla, CA, USA) was used for statistical analyses, using Kruskal-Wallis and Mann-Whitney. Kruskal-Wallis was used to compare the ability of the different *C*. *acnes* types and strains to induce platelet aggregation and activation. Mann Whitney was used to compare cfu/ml in the killing assay and to compare AS12 induced platelet aggregation and activation under different conditions. The co-localization of platelets and *C*. *acnes* in aggregates was calculated in NIS Elements (Nikon, Melville, NY, USA), using Pearson's correlation. p < 0.05 was considered significant.

## Results

### *C*. *acnes* can aggregate platelets

**Fig 1:**
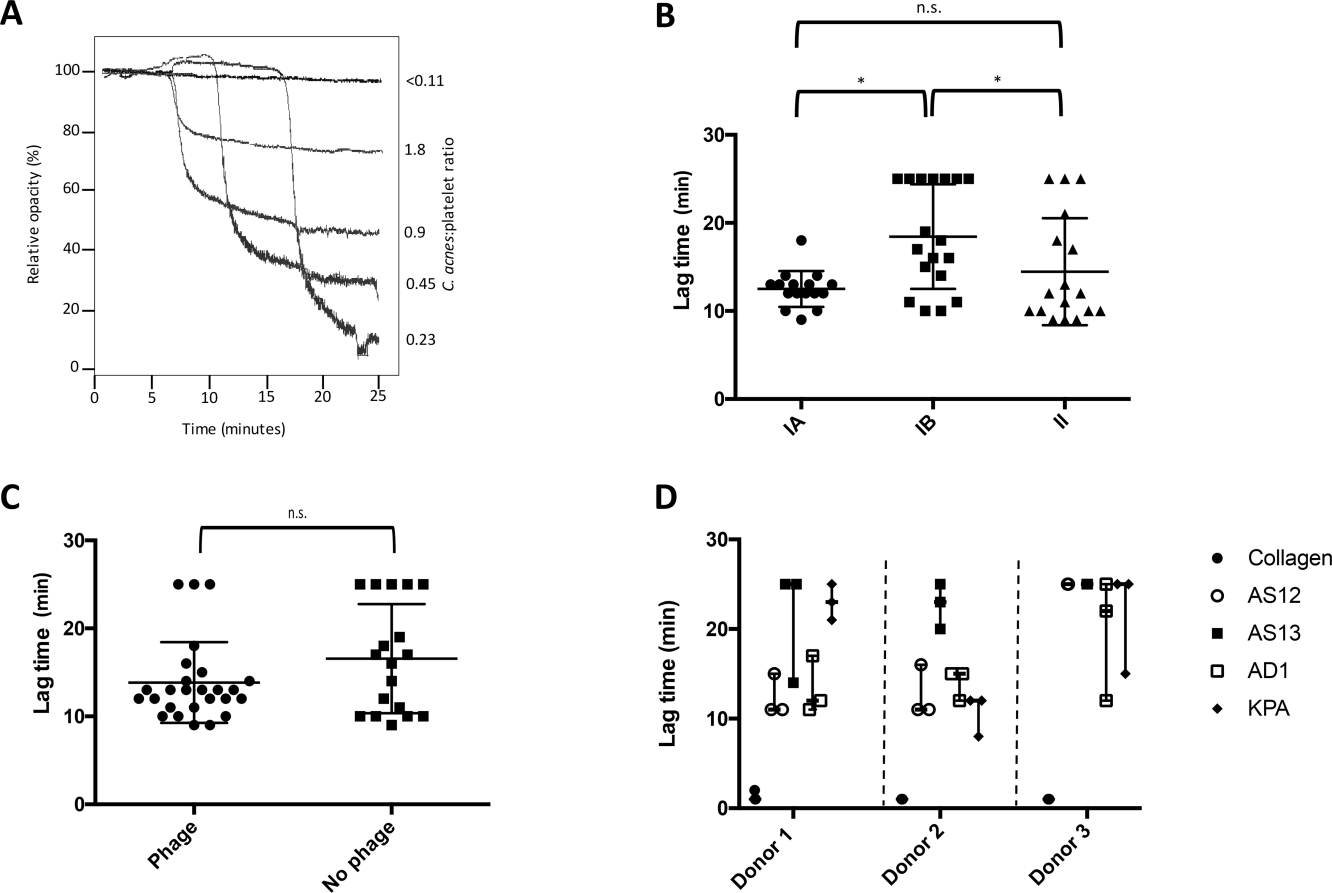
*C*. *acnes* aggregation of platelets is dose, type and donor-dependent. *C*. *acnes* isolate AS12 was incubated with platelets at different ratios (0.11:1–1.8:1; *C*. *acnes*:platelet) in plasma and aggregation measured using an aggregometer (Chronolog) (A). The ability of different types of *C*. *acnes* (B), presence of bacteriophages (C), or donors (D), to influence aggregation was investigated. Failure to induce aggregation is indicated by the maximum lag time (t = 25 min). Each dot in the figures represents one experiment (e.g. one bacterial strain). For phylotype and phage dependence, a single donor was used. All experiments were performed three independent times and are presented as medians with range where appropriate. Due to the non-parametric nature of the samples, Kruskal-Wallis (B, D), and Mann-Whitney (C) were used for statistical evaluation.

Despite causing low-grade chronic inflammation in plasma rich environments, the ability of *C*. *acnes* to activate and aggregate platelets has not been investigated. Using a skin isolate of *C*. *acnes* (AS12, phylotype II; [6]) we demonstrated that these bacteria can mediate platelet aggregation (Fig 1A). Platelet aggregation is reported both as the lag time to aggregation (*e*.*g*. time from addition of agonist to initiation of aggregation), and as the magnitude of aggregation, with the latter being reported as the percent of aggregation at the end of the experiment.

Other studies have indicated that the bacterium:platelet ratio influences the ability of platelets to aggregate [23]. Therefore, we incubated *C*. *acnes* at different concentrations with platelets and measured the aggregation. A *C*. *acnes*:platelet ratio of below 0.11 (*e*.*g*. ca 1 bacterium per 10 platelets) did not result in any measurable aggregation within 25 minutes, which was set as the upper time limit. However, higher ratio of bacteria:platelet (0.23–1.8) all resulted in aggregation within the set time frame. Higher doses of bacteria resulted in shorter lag times before aggregation (Fig 1A). The aggregation was not a non-specific clumping of cells as demonstrated by an inhibitory effect of the biochemical platelet inhibitor prostaglandin E (PGE_1_) (S1 Fig). By increasing the ratio bacteria:platelet, the lag time before aggregation was reduced. However, while the lag time was reduced with increasing bacterial concentrations, so was the relative drop in the opacity of the sample. This reduced opacity drop was not due to a lowered magnitude of platelet aggregation, as demonstrated by the inability of the physiological platelet agonist collagen to further lower the opacity in these samples (S2 Fig). Rather, the change in opacity drop can be derived to the fact that bacterial free PPP was used as a reference, not taking into account the impact in opacity that the increased bacteria resulted in. Bacteria grown to both exponential phase and stationary phase induced platelet aggregation with no significant difference. For experimental simplicity, we thus continued working with stationary phase bacteria.

### Platelet aggregation is *C*. *acnes* type-dependent

Historically, *C*. *acnes* has been divided into different types (IA, IB, and II) based on reactivity against specific antibodies [45]. More detailed genetic studies have recently been able to further subdivide the *C*. *acnes* population into several different sequence types [4]. The presence/absence of specific surface structures distinguishing the phylotypes from each other may result in different abilities to activate and aggregate platelets. To investigate this hypothesis, we studied the ability of recently typed isolates of *C*. *acnes* [6] to aggregate platelets. Type IA and II strains were significantly better at mediating platelet aggregation as analyzed by Kruskal Wallis tests (p = 0.034 and p = 0.045, respectively), while many type IB strains failed to initiate platelet aggregation (Fig 1B).

A common feature for many bacteria is the presence of prophage regions in their genome. These prophages are bacterial viruses, but often also encode virulence factors, increasing the pathogenicity of their host [46]. The presence of such bacterial viruses, or specifically certain phage proteins, has been demonstrated to mediate platelet aggregation in *Streptococcus mitis* [47,48]. The presence of bacteriophages in *C*. *acnes* has been well documented, with a significant lower abundance in phylotype IB strains [38]. However, the presence of bacteriophages in *C*. *acnes* [49], did not influence platelet aggregation (Fig 1C).

### Platelet aggregation is donor dependent

Our findings that different strains of *C*. *acnes* demonstrated distinct platelet aggregation responses, led us to investigate if also host differences (e.g. plasma proteins) would influence the platelet response. Four isolates of *C*. *acnes*, representing three phylotypes (type IA: AD1; type IB: KPA171202, AS13; type II: AS12) with different abilities to trigger platelet aggregation in one donor, were used to induce platelet aggregation in PRP from three individual donors. The three donors responded to the *C*. *acnes* isolates with different lag times before aggregation (p = 0.036). There was also a marked interindividual difference in lag times between single isolates (Fig 1D); a difference that remained even when non-aggregating strains were removed (S3 Fig). While certain strains (*e*.*g*. AS12 and AD1) induced platelet aggregation after ~10 minutes in two donors, they only induced platelet aggregation after 20+ minutes (AD1), or failed to induce aggregation in a third donor (Fig 1D). The difference between the strains is only significant in donor 2 (Kruskal Wallis, p = 0.025).

### Platelet activation is donor dependent

**Fig 2:**
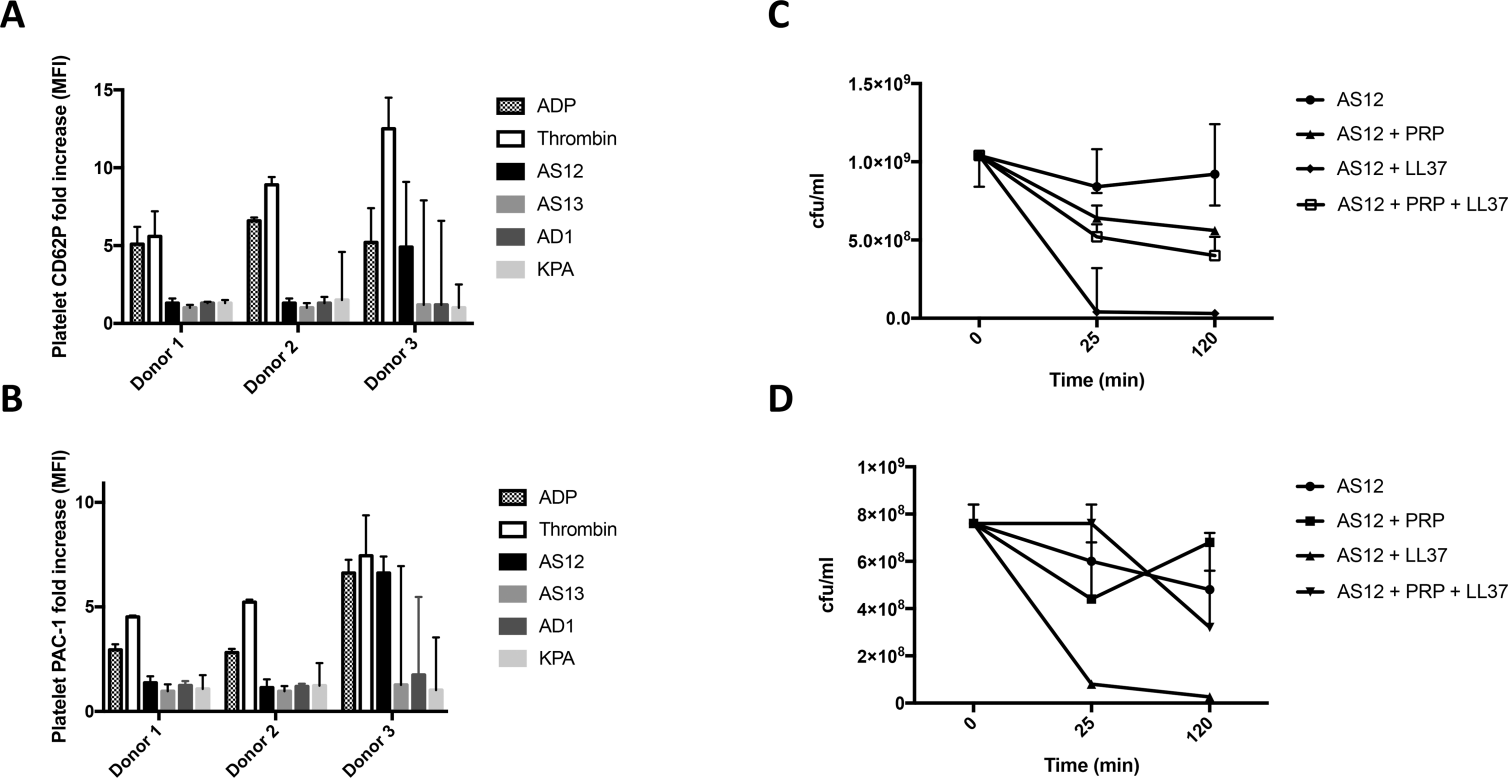
*C*. *acnes* activation of platelets is donor-dependent and does not affect bacterial viability. Platelet activation was measured by platelet surface-bound CD62P (A; FACS) and PAC-1 expression on platelets (B; FACS), in three different donors using four strains of *C*. *acnes* (AS12, AS13, AD1, KPA171202), and the difference between the donors was investigated using Kruskal Wallis (A; p = 0.0076, B; p = 0.0132). The ability of *C*. *acnes* strain AS12 to survive in the presence of PRP with or without LL37 was measured at three different time points (0, 25, and 120 minutes after mixture) after vortex (C) or combining vortex and sonication (D) before serial dilution and cfu counting. Bacterial count did not change significantly over time as assessed by Kruskal Wallis (n.s.). In all activation experiments, collagen, ADP and thrombin were used as controls where applicable, and PRP and HEPES buffer as references. All experiments were performed three independent times and presented as medians with range.

Platelet activation is generally followed by secretion of granules, and surface attachment of the platelet alpha granule protein CD62P and upregulation of PAC-1. Donor 3 had a higher fold increase of CD62P than donor 1 and 2 (Kruskal Wallis, p = 0.008)(Fig 2A). A similar trend could be visualized by studying the fold increase of PAC-1, indicating activated platelets (Kruskal Wallis, p = 0.013)(Fig 2B). Strain AS12 induced an increase in CD62P and PAC-1 in donor 3, however, there was no significant difference in the platelet activation by the different strains. Of note, the two donors responding with a lower fold increase of CD62P/PAC-1, were potent in causing aggregation of platelets (Fig 1D). S4 Fig displays representative raw flow cytometry data where gating of a platelet population in platelet-rich plasma and the PAC-1 and CD62P signals on the surface of the platelets in that population are visualized (S4 Fig).

### Platelet aggregation does not kill *C*. *acnes*

To further investigate the biological relevance of platelet aggregation for the bacterium:host interaction, we incubated *C*. *acnes* with PRP and measured the bacterial survival at different time points representing the time of inoculation (t = 0 min), aggregation (t = 25 min), and prolonged interaction (t = 120 min). Aggregation was confirmed macroscopically for all samples at time points 25 and 120 min, indicating that the platelets had aggregated. Samples were gently sonicated to disrupt either bacterium:bacterium interactions, or platelet aggregates to accurately count live bacteria. Sonication resulted in similar levels of free bacteria as vortex treated samples, but was too gentle to induce bacterial lysis (Fig 2C and 2D)). While addition of the platelet secreted antimicrobial peptide LL-37 induced killing of *C*. *acnes*, no killing or reduced viability of *C*. *acnes* by platelet aggregation itself could be observed (Fig 2C and 2D). Rather, platelet aggregation served as a protective barrier against LL-37, reducing its antimicrobial activity (Fig 2C and 2D). The changes were however not statistically significant.

### Aggregation is dependent on plasma components

**Fig 3:**
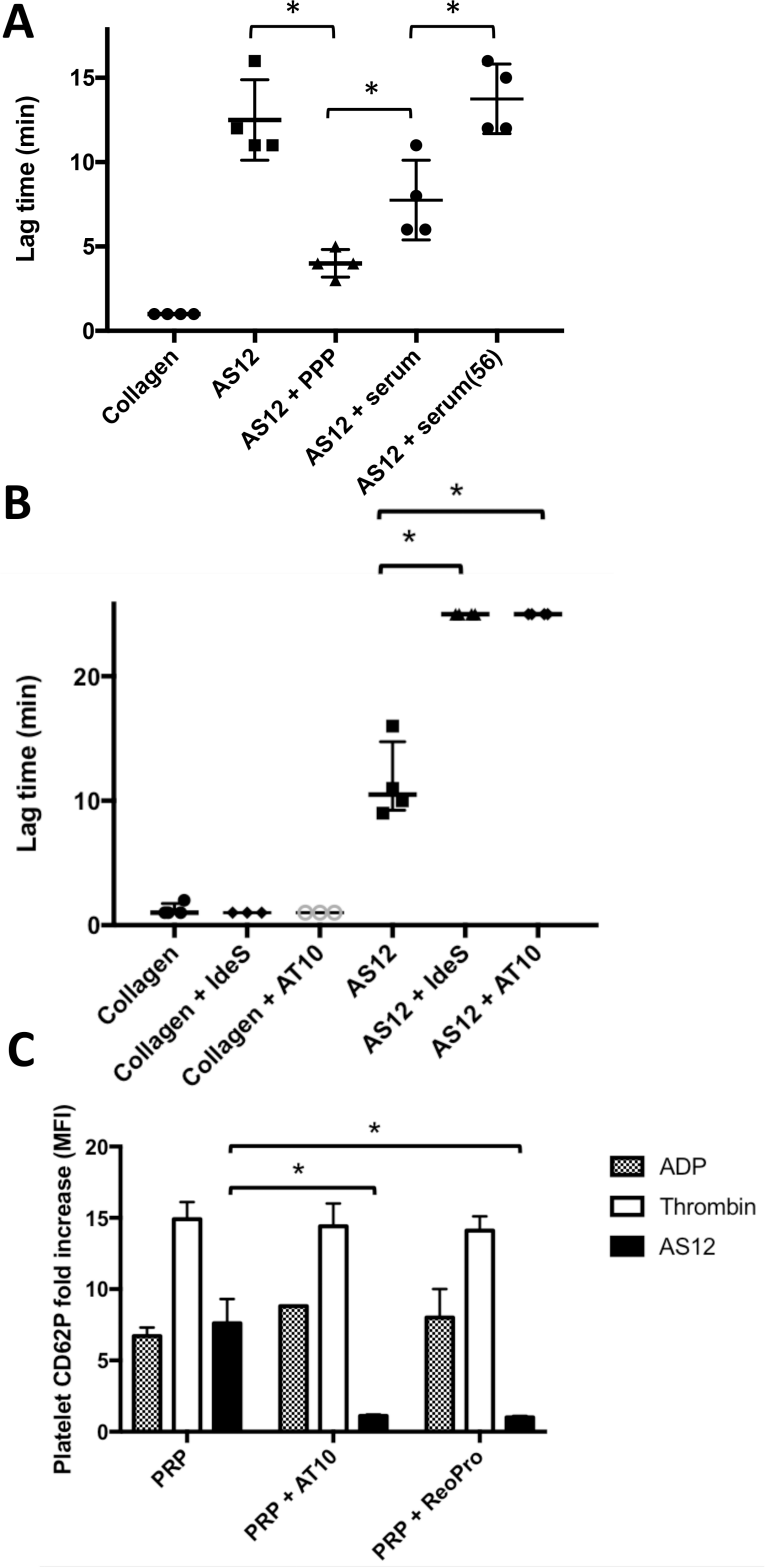
IgG and fibrinogen are critical mediators for *C*. *acnes* platelet aggregation. Pretreatment of *C*. *acnes* strain AS12 with plasma (PPP) or serum significantly reduces the lag time before aggregation of PRP (A; p = 0.03), while pretreatment with heat-inactivated serum does not reduce the lag time. Inhibition of IgG-mediated aggregation of platelets through pre-incubation of PRP with IdeS and AT10 resulted in significant inhibition in aggregation (B; p = 0.03). IdeS will inhibit IgG mediated signaling through specific cleavage of IgG in the hinge region, while AT10 mediates its effect through FcRγIIA antagonist properties. PRP was pre-treated with Abciximab in order to investigate the importance of fibrinogen in platelet aggregation of *C*. *acnes* stimulated platelets (C; CD62P; p = 0.04). All experiments were performed four independent times and presented as medians with range and analyzed using Mann Whitney.

The observed differences in activation and aggregation between donors, and the relatively long lag-time before aggregation, could indicate deposition of plasma proteins on the bacterial surface, facilitating aggregation of the platelets. To investigate this, we used *C*. *acnes* strain AS12 due to its high ability to induce aggregation. Pre-incubation of bacteria with plasma led to a significantly shorter lag-time as compared to non-treated bacteria (p = 0.03)(Fig 3A). To elucidate the role of complement compounds in the platelet aggregation the bacteria were pre-incubated with serum or serum that was heat inactivated at 56° C to inhibit complement activation. Pre-incubation of bacteria with serum led to a significantly shorter lag-time as compared to non-treated bacteria (p = 0.03) but a significantly longer lag-time as compared to bacteria pre-incubated with plasma (p = 0.03)(Fig 3A). Pre-incubation of bacteria with heat-inactivated serum led to a significantly longer lag-time as compared to bacteria pre-incubated with serum (p = 0.03), equivalent to non-treated bacteria (Fig 3A). To investigate the role of IgG, we determined the presence of *C*. *acnes*-specific antibodies in several donors. All donors had antibodies directed towards the four investigated strains of *C*. *acnes*. Individual donors had similar antibody titers to all *C*. *acnes* strains, however, there was an interindividual difference in *C*. *acnes* titers (p<0.001)(S5 Fig). Presence of high anti-*C*. *acnes* antibody titers did not *per se* correlate with high ability to cause aggregation. Blocking of IgG-mediated aggregation of platelets, either through hydrolysis of IgG by IdeS-treatment (cleaves IgG in the hinge, generating a Fab- and Fc-fragment)[40], or by blocking of FcγRIIA through AT10, completely abolished platelet aggregation mediated by bacteria but not controls (Fig 3B and 3C)(p = 0.03 and p = 0.03, respectively), indicating that IgG is a critical, though not the only, component for *C*. *acnes* aggregation of platelets.

Other than IgG, fibrinogen has been indicated as one of the main components involved in platelet activation and aggregation in response to bacteria. Blocking of signaling through the glycoprotein IIb/IIIa, functioning as a fibrinogen receptor on the platelet surface, using the receptor antagonist Abciximab, significantly inhibited platelet activation in response to *C*. *acnes* (p = 0.03), while not affecting activation by ADP or thrombin (Fig 3C).

### Plasma protein deposition on the surface of *C*. *acnes*

**Fig 4:**
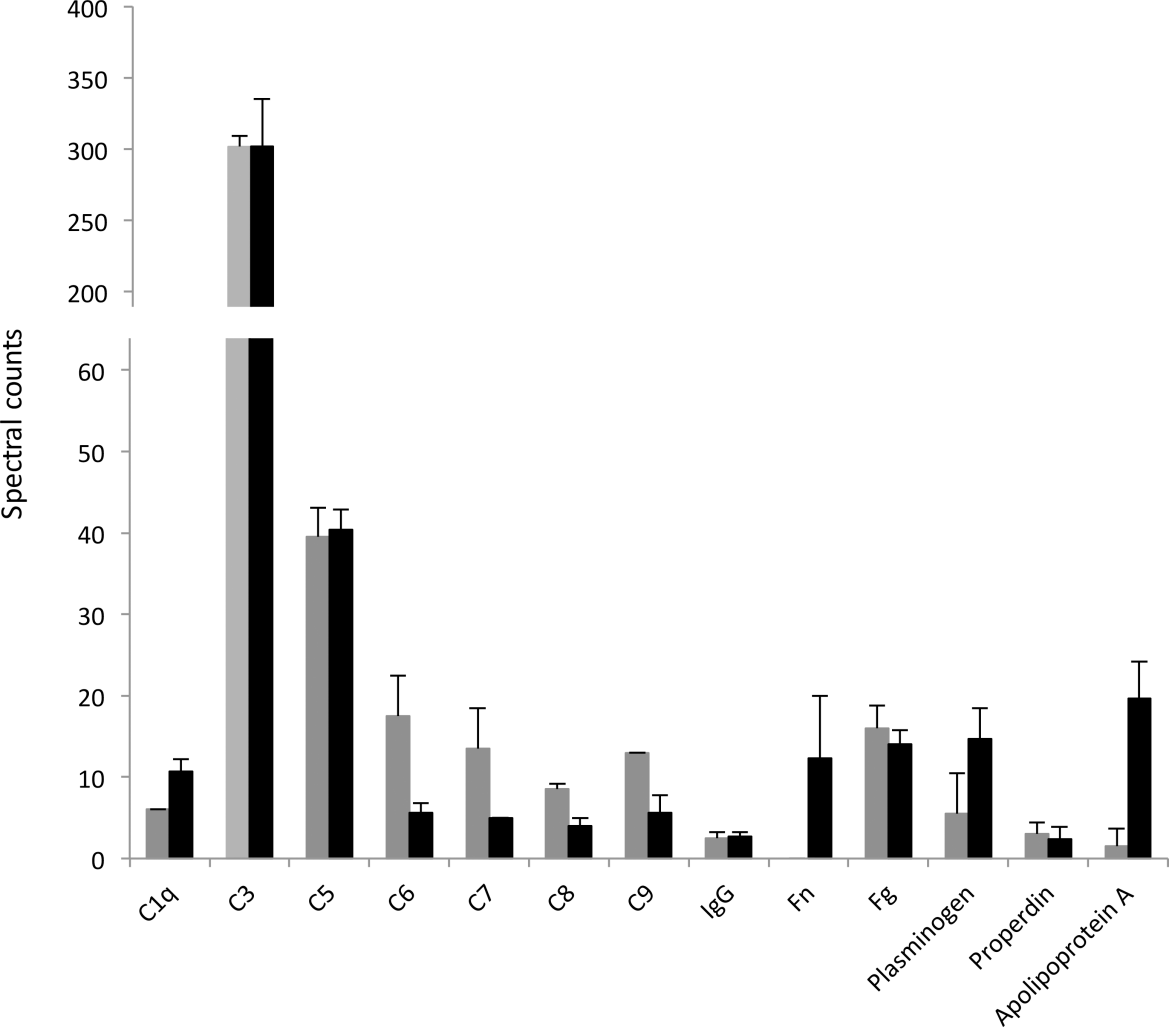
Mass spectrometry analysis of abundant plasma proteins deposited on *C*. *acnes*. *C*. *acnes* strains KPA171202 (black) and AS12 (gray) were grown in the presence of 2% plasma after which they were thoroughly washed, adherent proteins released by trypsin digestion, and analyzed using Shotgun mass spectrometry. Only the most abundant identified proteins are displayed and presented as means (± SD) from three biological replicates.

Having demonstrated that plasma proteins in general, and IgG, fibrinogen, and complement specifically, were critical components for mediating aggregation of platelets by *C*. *acnes*, we sought to investigate if we could identify these, and other, plasma proteins deposited on the surface of *C*. *acnes* using mass spectrometry. The two *C*. *acnes* strains investigated were decorated with IgG, fibrinogen and activated complement factors (Fig 4). Only strain KPA171202 was able to bind fibronectin, plasminogen, and apolipoprotein A to a much higher degree than AS12. Due to the different abilities of peptides to fly in the mass spectrometer, and thus be detected and quantified, peptide abundance as measures by mass spectrometry does not necessarily equal an absolute quantity. For that reason, comparison of peptide abundance between different proteins in this setting (e.g. complement factors and IgG) is not valid. However, comparison of the same protein between different strains (e.g. AS12 vs KPA171202) is.

The distinct plasma binding profiles of the two investigated *C*. *acnes* strains point towards the presence of cell-wall anchored strain-specific surface receptors for these proteins in *C*. *acnes*. The identification of these receptors, or a further detailed study of the binding preferential of plasma proteins to *C*. *acnes* is however beyond the scope of this study.

### Visualization of *C*. *acnes*-platelet interactions using fluorescence microscopy

**Fig 5:**
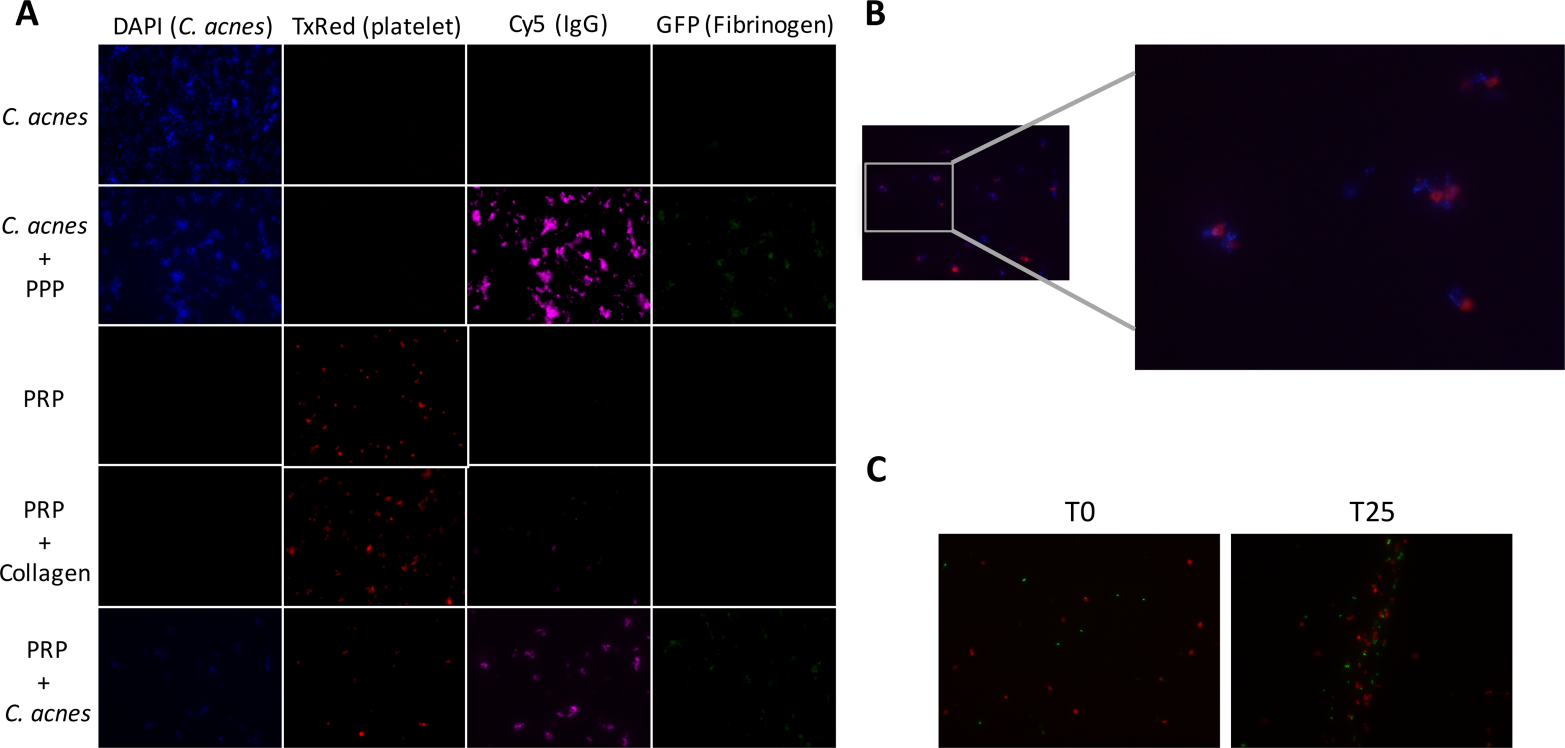
Fluorescence microscopy indicates co-localization of *C*. *acnes* and platelets. *C*. *acnes* isolate AS12 was incubated with PPP or PRP, and fluorescent probes against the bacterium (DAPI), platelets (TxRed; Alexa Fluor 594), IgG (Cy5; Alexa Fluor 647), and fibrinogen (GFP; FITC), used to visualize the presence and localization of targets. PBS, PPP and PRP alone were used as negative controls, and collagen I as a positive control for platelet aggregation (A). Co-localization of *C*. *acnes* and platelets was analyzed by merging the signal for DAPI (*C*. *acnes*) and TxRed (platelets) (B). The co-localization of *C*. *acnes* (Oregon Green) and platelets (TxRed) after 25 minutes (T25) and at starting point (T0) was compared and analyzed using Pearson's correlation (p = 0.017)(C). All micrographs are at 40x magnification.

To further investigate, and visualize, a direct interaction between *C*. *acnes*, plasma proteins, and platelets, we used fluorescence microscopy. In concurrence with the mass spectrometry data, we could identify high levels of IgG deposited on the bacteria, as well as fibrinogen (Fig 5A). Furthermore, *C*. *acnes* and platelets could be individually labeled (Fig 5A), and demonstrated to co-localize within cellular aggregates (p = 0.017)(Fig 5B and 5C).

## Discussion

*C*. *acnes* is a bacterium that historically has been studied for its immunomodulatory effects, ranging from anti-tumor effects [16,50], and T-cell population dynamics regulation [51]; to acne [52], joint prosthesis inflammations [53], and prostate cancer [54]. With the recent increase of isolated, or detectable, *C*. *acnes* from biofilms formed on medical implants or catheters [55], the importance of this bacterium in pathogenesis other than its association with acne has been raised [5]. However, mechanistic insights into the interaction of *C*. *acnes* and distinct blood cells have been lacking.

It has been argued that all species of bacteria should be able to induce platelet aggregation, and the microbiota more so than pathogens, should antibodies directed towards them exist in the circulation [56]. Being part of the skin microbiota on the vast majority of the population, it has been shown that most individuals have antibodies against *C*. *acnes* in the bloodstream, even though the titer can vary [57,58]. These antibodies demonstrate specificity against a few selected proteins, however, these proteins are apparently evenly distributed amongst all the different phylotypes of *C*. *acnes*, indicating that there is no discrepancies in antibody-binding between the different types [57]. This is of particular interest since other studies have demonstrated a phylotype and growth-condition specific proteome amongst *C*. *acnes* isolates [59]. Our data support this first conclusion, demonstrating similar antibody titers against distinct types of *C*. *acnes* within single individuals, whilst varying between individuals. Even though antibodies directed towards the bacterium were present, several isolates failed to induce activation and aggregation of platelets (Fig 1B). This was significantly pronounced in type IB strains. It may be that despite antibody binding to IB strains, IgG is not able to crosslink (e.g. their antigen could be localized on small islands on the surface), and would thus not be able to initiate a complement cascade and platelet binding/aggregation. The complement deposited on the surface of *C*. *acnes*, as detected by mass spectrometry, would thus be activated through the alternative or the MBL pathway. Further experiments are needed to fully understand the discrepancy between the phylotypes. The inability of *C*. *acnes* strains to mediate aggregation of platelets despite the presence of anti-*C*. *acnes* IgG could also be due to other limiting factors, including fibrinogen and complement. One factor in itself is not enough to initiate the aggregation, but they function in concert with each other. Therefore, the removal of one will abolish aggregation, but the presence of one does not *per se* indicate that aggregation will take place.

Type IB strains have earlier been demonstrated to have distinct properties as compared to type IA and II, in terms of carriage of bacteriophages [38], expression of CAMP factors [60], and genetic elements [61]; and we show here a difference in interaction with plasma proteins (*e*.*g*. fibronectin). However, the molecular mechanism(s) behind these phenotypic oddities have not been carefully elucidated. It is clear, based on our data, that the presence of IgG is but one critical factor necessary for the aggregation of platelets by *C*. *acnes*, but may not necessarily be the limiting factor in generating platelet aggregation, since both platelet-aggregating and non-aggregating strains of *C*. *acnes* had similar degrees of IgG opsonization. Even though *C*. *acnes* type IB is not as abundant as type IA on the skin, it is still a common skin bacterium. However, the abundance of type IA on the skin may explain why the immune system is more adapt in responding to those isolates through aggregation, constantly being stimulated by these strains [10,62].

Many bacteria rely on the presence of IgG and fibrinogen for the activation and subsequent aggregation of platelets [56]. *C*. *acnes* was recently shown to have specific fibrinogen-binding proteins [63], and has earlier been shown to secrete proteins capable of interacting with TLR2-receptors [64], possibly partaking in the aggregation of platelets. It is however currently unknown as to what percentage of clinical strains carry this gene, as well as if it is involved in platelet aggregation. Fibrinogen binding has proven critical for other skin bacteria to induce thrombosis, including both ClfA and ClfB in *Staphylococcus aureus* [65], and SdrG in *Staphylococcus epidermidis* [66], being prevalent in a majority of clinical isolates [67]. In many aspects, *C*. *acnes* aggregation of platelets seems more similar to that of *Aerococcus urinae*, recently demonstrated being able to cause aggregation of platelets [23]. Both bacteria rely on a high bacterium:platelet ratio for initiation of platelet aggregation, and an indirect activation/aggregation of platelets based on presence of IgG and fibrinogen [23]. Other bacteria (*e*.*g*. *Streptococcus pyogenes*), only demand a low number of bacteria to cause aggregation, occurring within 30–90 seconds [20], as compared to 10–20 minutes for *C*. *acnes* and *A*. *urinae* [23]; demonstrating a difference in aggregation response for different bacteria.

During analysis of the donor-dependency in platelet responses, we found a discrepancy in the results regarding donor activation (CD62P, PAC-1) and aggregation. However, all donors responded in either one or both the assays. The assays are only partly comparable because flow cytometry investigates an early stage platelet response and does not reflect aggregation, while aggregation in the Chrono Log only measures complete platelet aggregation and does not reflect the early platelet response. Further, during this analysis it became clear that there is a biological variance allowing for some variation in response to bacteria. Donor 3 was noticeably a weak initiator of platelet aggregation at two time-points, but initiated aggregation in two strains at the third occasion (Fig 1B). Donor 1 and 2 also showed variation in response to stimuli based on the day of the experiment, though less significant. Due to the multifactorial interaction, many factors may participate in the slightly varied responses, be it concentration of plasma proteins (fibrinogen) which are known to vary in particular amongst fertile women (e.g. menstruation) [68], or be it expression of bacterial surface proteins. Nevertheless, this clearly shows a donor dependent response.

Mass spectrometry data as well as fluorescence microscopy both support the presence of IgG, fibrinogen, and complement on the surface of *C*. *acnes*; both on type II (AS12) and type IB (KPA171202). Using mass spectrometry we could further identify several other plasma proteins at the surface of *C*. *acnes*, eg. apolipoprotein A and plasminogen, currently not known to be involved in platelet aggregation. These proteins displayed a strain-specific binding. However, the biological relevance of this is currently unknown, making it an interesting future area of investigation. The lack of aggregation in several type IB isolates should thus not be due to a lack of plasma protein deposition on the surface, or reduced opsonization of the targets. A recent study highlighted the differences in surface and secreted proteomes of *C*. *acnes* types, and distinct immune responses towards these strains from PBMCs [69], suggesting that different types of *C*. *acnes* may also induce different responses from platelets, as seen in our study.

The presence of complement on the surface of *C*. *acnes* is also noteworthy. Complement is responsible for opsonization of bacteria, and as such should be identified on pathogens incubated with plasma. However, complement is also a mediator of activation/aggregation of platelets, usually in combination with IgG [46,55]. It has been suggested that signaling through IgG/FcRIIa in combination with complement and complement receptors on the surface of platelets can induce a slow activation of platelets [46]. Other bacteria, including *A*. *urinae* has demonstrated a dependence of complement, beyond their dependence on IgG and fibrinogen for platelet aggregation [15]. The prolonged lag-time after incubation of *C*. *acnes* with heat-inactivated serum, and the accumulation of complement compounds on the bacterial surface after incubation with plasma, observed in this study suggests that complement plays a role also in platelet aggregation induced by *C*. *acnes*.

Even though the platelet response towards *C*. *acnes* leads to aggregation, the biological significance of this for the survival of *C*. *acnes* is debatable. Bacteria are supposedly contained by the platelets and delivered high concentrations of bacteriocidal substances from the activated platelets. Certain pathogens, including *S*. *pyogenes* have evolved means to survive this, and even take advantage of this containment for improved survivability in blood before releasement from the aggregate [20]. Seemingly, *C*. *acnes* can induce aggregation of platelets without prior activation of the platelets, leading to an entrapment, but not killing, of the bacteria. Interestingly, even addition of high concentrations of LL-37 (250 μM) to the mixture did not affect the viability of the bacteria but was antibacterial in the absence of PRP. While LL-37 has a slightly lowered antibacterial activity in serum as compared to PBS, 20 μM LL-37 has been reported to still have a significant antibacterial effect [70]. Thus, we speculate that *C*. *acnes* can induce platelet aggregation for protection against serum proteins (*e*.*g*. LL-37), facilitating survival in this harsh environment. *C*. *acnes* is noticeably difficult for the immune system to handle, and has recently been suggested to survive intracellularly within macrophages and osteoblasts [9,71]. Of more importance, *C*. *acnes* has been reported to withstand most of the effects of lysozyme, PMNs, monocytes, and their released contents, including MPOs [72]. Whether this partly relates to the ability of *C*. *acnes* to efficiently reduce free oxygen radicals through its secreted antioxidant RoxP remains to be elucidated [73]. However, based on our data it is clear that platelet aggregation by *C*. *acnes* is multifactorial, including several plasma proteins (IgG and fibrinogen) as well as possibly specific surface antigens, less commonly exposed (or expressed at a lower ratio) in type IB strains.

The study has a few limitations that should be noted. Firstly, in most scenarios when *C*. *acnes* will be in contact with platelets, it will mainly be in either a low infection dose (e.g. in platelet concentrates, skin wounds, etc), such that no platelet aggregation will be initiated, or as part of a biofilm. Initial experiments indicate that platelets will recognize a biofilm and aggregate upon contact with *C*. *acnes* biofilms, however more research is needed to further investigate this. Further, while not necessarily being a limitation, the partly activation-independent platelet aggregation mediated by *C*. *acnes* is rather unusual, since most platelet aggregations are initiated by an initial activation [24]. There are however reports of platelet aggregation in the absence of activation due to an increased stress of the platelets [74]. The mechanism of *C*. *acnes* eliciting an aggregation in absence of activation remains to be elucidated. Thus, while providing evidence of a *C*. *acnes*-platelet interaction, being mediated by common platelet-activating proteins, leading to aggregation, it is clear that this particular interaction is more than meets the eye, and further research will be needed to investigate these interactions.

## Supporting information

S1 Fig Platelet aggregation is mediated by bacteria. Platelet rich plasma (PRP) was incubated with collagen (5 μg/ml) as a positive control (A), and with *C*. *acnes* (1x10^8^ cfu/ml) and PBS (B; blue), or *C*. *acnes* (1x10^8^ cfu/ml) and prostaglandin E (1 μM)(B; black). Aggregation was measured using a platelet aggregometer (ChronoLog) and analyzed using AggroLink.
(PDF)

S2 Fig Percent platelet aggregation is affected by the overall opacity of the sample. Platelet rich plasma (PRP) was incubated with collagen (5 μg/ml) as a positive control (A; blue), and with *C*. *acnes* (1x10^8^ cfu/ml) and PBS (A; black). To PRP incubated with a high concentration of *C*. *acnes* (4x10^8^ cfu/ml), PBS or collagen (5 μg/ml) was added after 16 minutes (B; blue and black, respectively). The immediate drop is due to technical reasons when adding the reagents (*e*.*g*. PBS and collagen). Aggregation was measured using a platelet aggregometer (ChronoLog) and analyzed using AggroLink.
(PDF)

S3 Fig *C*. *acnes* donor- and strain-dependent aggregation of platelets with non-aggregating strains excluded. *C*. *acnes* isolates AS12, AS13, AD1 and KPA171202 were incubated with platelets in plasma and aggregation measured using an aggregometer (Chronolog). Failure to induce aggregation (t>25 min) resulted in removal of that strain. Each dot in the figures represents one experiment (e.g. one bacterial strain). All experiments were performed three independent times and are presented as medians with range where appropriate.
(PDF)

S4 Fig *C*. *acnes* mediated platelet activation detected using flow cytometry. Representative figure demonstrating the gating of a platelet population in platelet-rich plasma and the detection of PAC-1 and CD62P on the surface of the platelets in that population.
(PDF)

S5 Fig Concentration of anti-*C*. *acnes* antibodies varies between individuals. Serum from three donors was incubated with 4 different strains of *C*. *acnes* and deposition of IgG detected through flow cytometry and reported as fold increase of median fluorescence intensity / platelet (anti-IgG FITC).
(PDF)

S1 Table *C*. *acnes* strains used with corresponding phylotype and prophage information. (XLSX)
