# Supplementary material for: Platelet activation and aggregation by the opportunistic pathogen Cutibacterium (Propionibacterium) acnes
Source: PLoS One. 2018 Jan 31;13(1):e0192051. doi: 10.1371/journal.pone.0192051 (PMC5792000; doi:10.1371/journal.pone.0192051)
Supplement: S1 Fig — Platelet rich plasma (PRP) was incubated with collagen (5 μg/ml) as a positive control (A), and with C. acnes (1x10^8^ cfu/ml) and PBS (B; blue), or C. acnes (1x10^8^ cfu/ml) and prostaglandin E (1 μM)(B; black). Aggregation was measured using a platelet aggregometer (ChronoLog) and analyzed using AggroLink. (PDF) [file pone.0192051.s001.pdf]

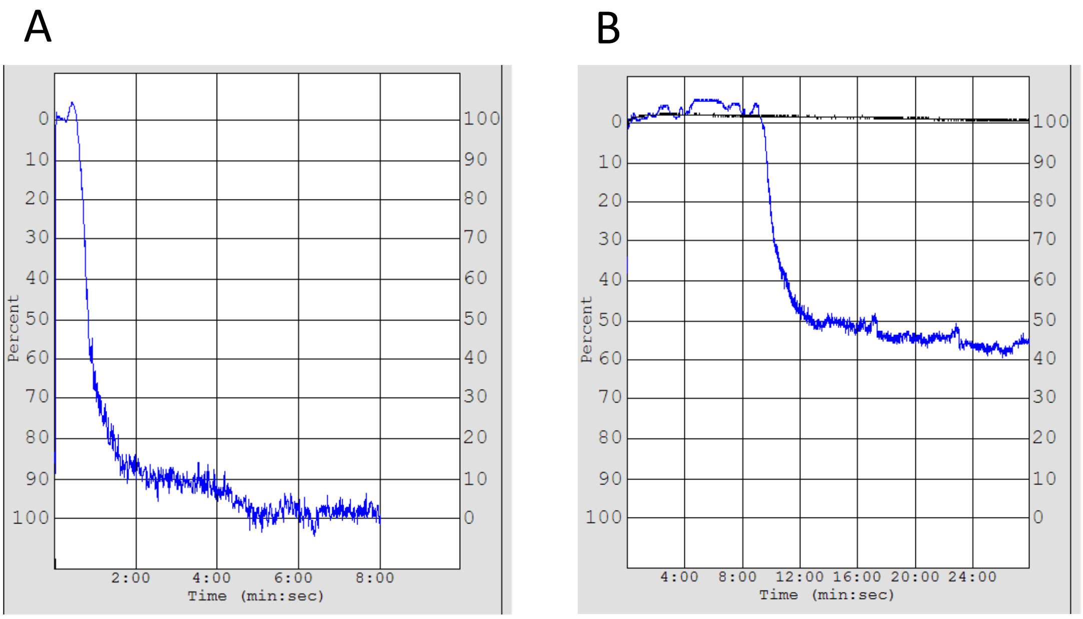

S1 Fig. Platelet aggregation is mediated by bacteria. Platelet rich plasma (PRP) was incubated with collagen (5 µg/ml) as a positive control (A), and with *C. acnes* (1x10^8 cfu/ml) and PBS (B; blue), or *C. acnes* (1x10^8 cfu/ml) and prostaglandin E (1 µM)(B; black). Aggregation was measured using a platelet aggregometer (ChronoLog) and analyzed using AggroLink.
