# Supplementary material for: Platelet activation and aggregation by the opportunistic pathogen Cutibacterium (Propionibacterium) acnes
Source: PLoS One. 2018 Jan 31;13(1):e0192051. doi: 10.1371/journal.pone.0192051 (PMC5792000; doi:10.1371/journal.pone.0192051)
Supplement: S2 Fig — Platelet rich plasma (PRP) was incubated with collagen (5 μg/ml) as a positive control (A; blue), and with C. acnes (1x10^8^ cfu/ml) and PBS (A; black). To PRP incubated with a high concentration of C. acnes (4x10^8^ cfu/ml), PBS or collagen (5 μg/ml) was added after 16 minutes (B; blue and black, respectively). The immediate drop is due to technical reasons when adding the reagents (e.g. PBS and collagen). Aggregation was measured using a platelet aggregometer (ChronoLog) and analyzed using AggroLink. (PDF) [file pone.0192051.s002.pdf]

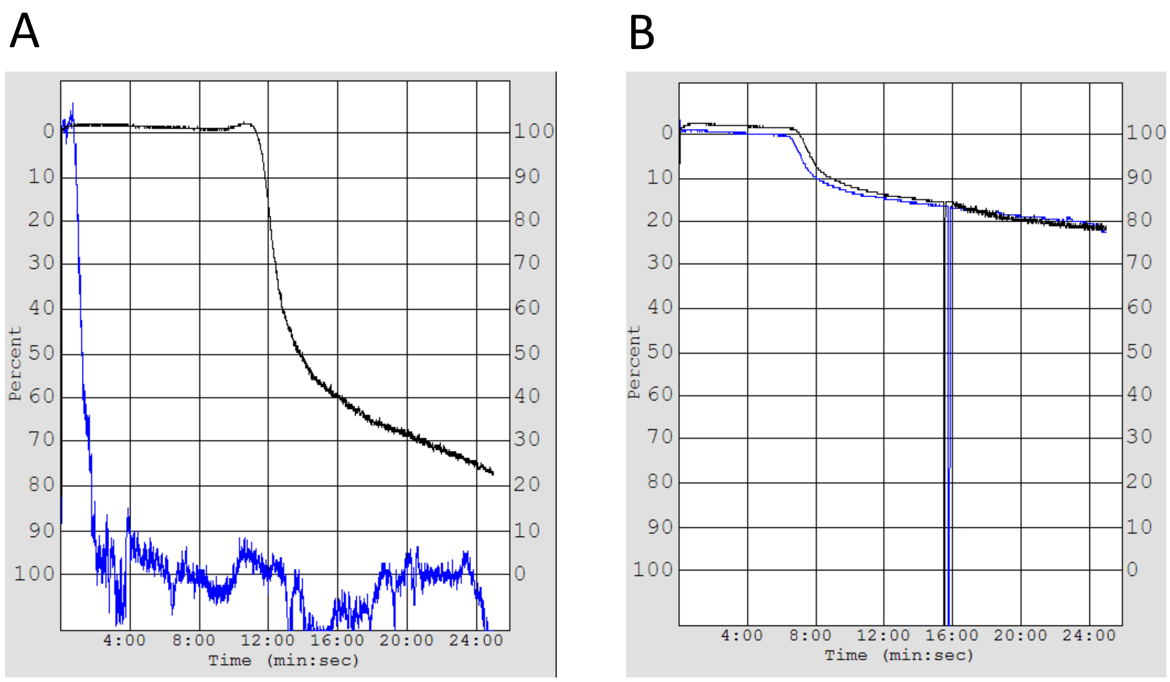

S2 Fig. Percent platelet aggregation is affected by the overall opacity of the sample. Platelet rich plasma (PRP) was incubated with collagen (5 µg/ml) as a positive control (A; blue), and with *C. acnes* ($1x10^8$ cfu/ml) and PBS (A; black). To PRP incubated with a high concentration of *C. acnes* ($4x10^8$ cfu/ml), PBS or collagen (5 µg/ml) was added after 16 minutes (B; blue and black, respectively). The immediate drop is due to technical reasons when adding the reagents (*e.g.* PBS and collagen). Aggregation was measured using a platelet aggregometer (ChronoLog) and analyzed using AggroLink.
