# Supplementary material for: Platelet activation and aggregation by the opportunistic pathogen Cutibacterium (Propionibacterium) acnes
Source: PLoS One. 2018 Jan 31;13(1):e0192051. doi: 10.1371/journal.pone.0192051 (PMC5792000; doi:10.1371/journal.pone.0192051)
Supplement: S3 Fig — C. acnes isolates AS12, AS13, AD1 and KPA171202 were incubated with platelets in plasma and aggregation measured using an aggregometer (Chronolog). Failure to induce aggregation (t>25 min) resulted in removal of that strain. Each dot in the figures represents one experiment (e.g. one bacterial strain). All experiments were performed three independent times and are presented as medians with range where appropriate. (PDF) [file pone.0192051.s003.pdf]

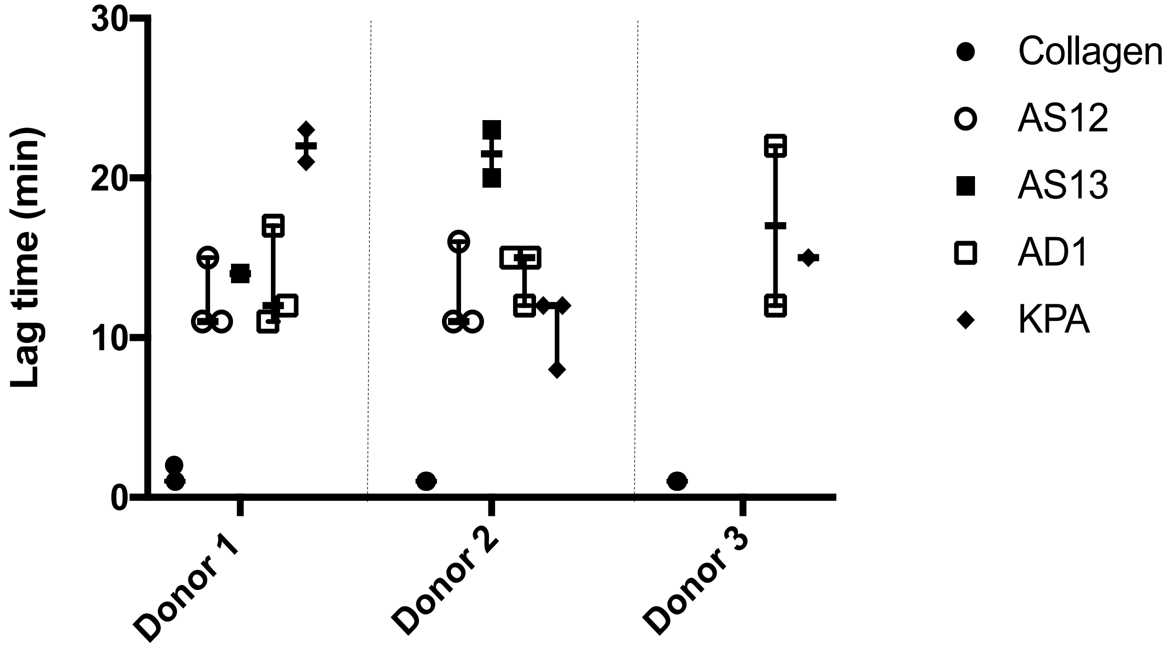

S3 Fig. *C. acnes* donor- and strain-dependent aggregation of platelets with non-aggregating strains excluded. *C. acnes* isolates AS12, AS13, AD1 and KPA171202 were incubated with platelets in plasma and aggregation measured using an aggregometer (Chronolog). Failure to induce aggregation (t>25 min) resulted in removal of that strain. Each dot in the figures represents one experiment (e.g. one bacterial strain). All experiments were performed three independent times and are presented as medians with range where appropriate.
