# Supplementary material for: Platelet activation and aggregation by the opportunistic pathogen Cutibacterium (Propionibacterium) acnes
Source: PLoS One. 2018 Jan 31;13(1):e0192051. doi: 10.1371/journal.pone.0192051 (PMC5792000; doi:10.1371/journal.pone.0192051)
Supplement: S4 Fig — Representative figure demonstrating the gating of a platelet population in platelet-rich plasma and the detection of PAC-1 and CD62P on the surface of the platelets in that population. (PDF) [file pone.0192051.s004.pdf]

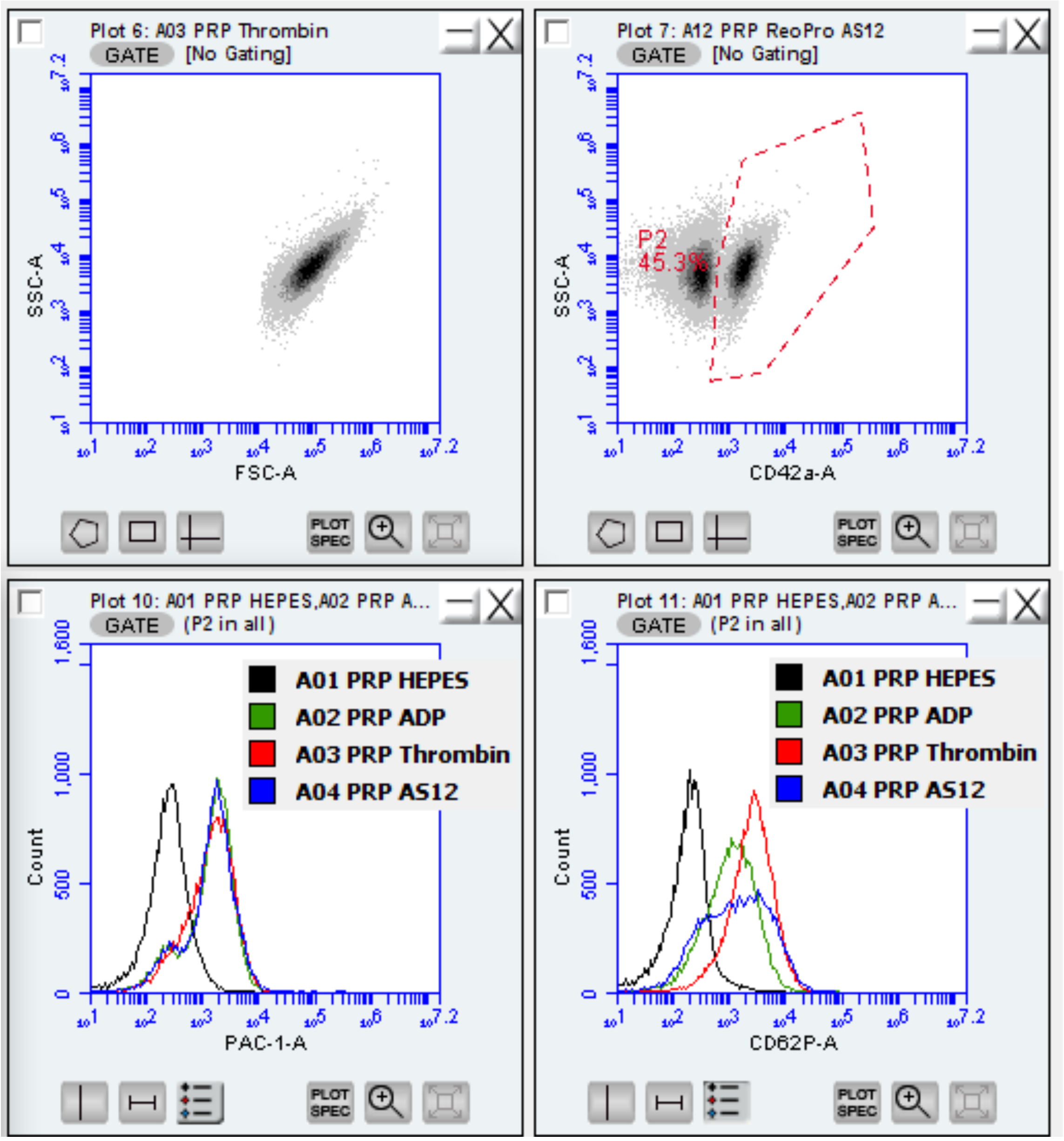

S4 Fig. *C. acnes* mediated platelet activation detected using flow cytometry. Representative figure demonstrating the gating of a platelet population in platelet-rich plasma and the detection of PAC-1 and CD62P on the surface of the platelets in that population.
