# Supplementary material for: Platelet activation and aggregation by the opportunistic pathogen Cutibacterium (Propionibacterium) acnes
Source: PLoS One. 2018 Jan 31;13(1):e0192051. doi: 10.1371/journal.pone.0192051 (PMC5792000; doi:10.1371/journal.pone.0192051)
Supplement: S5 Fig — Serum from three donors was incubated with 4 different strains of C. acnes and deposition of IgG detected through flow cytometry and reported as fold increase of median fluorescence intensity / platelet (anti-IgG FITC). (PDF) [file pone.0192051.s005.pdf]

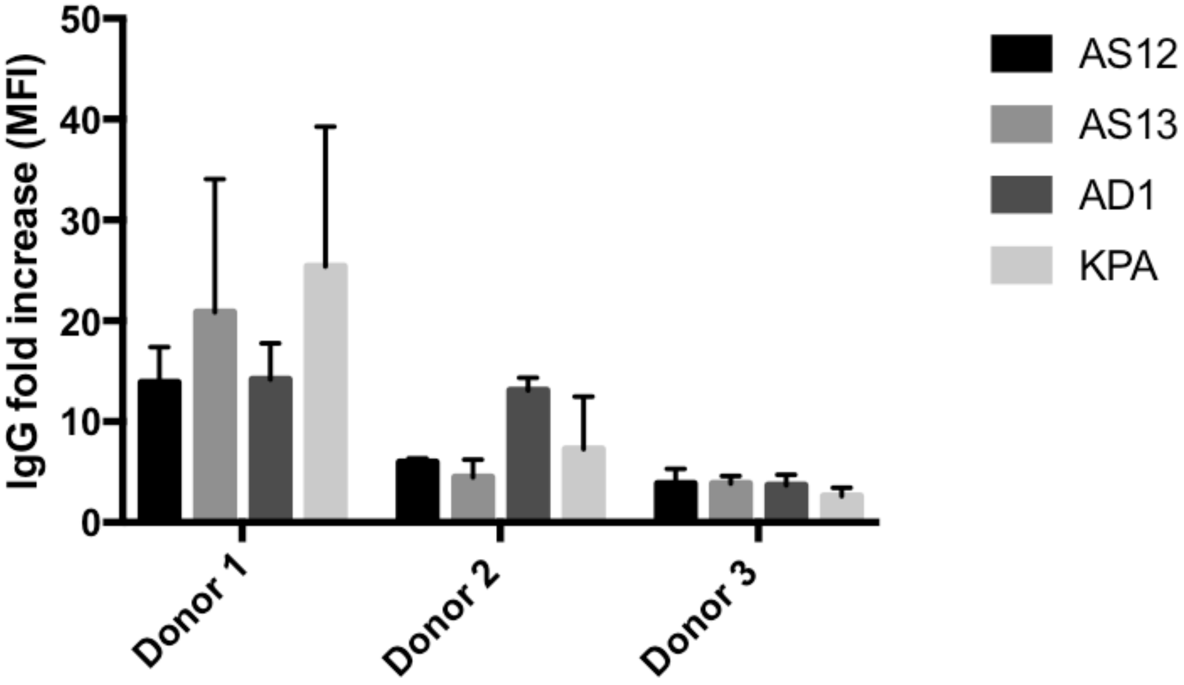

S5 Fig. Concentration of anti-*C. acnes* antibodies varies between individuals. Serum from three donors was incubated with 4 different strains of *C. acnes* and deposition of IgG detected through flow cytometry and reported as fold increase of mean fluorescence intensity / platelet (anti-IgG FITC).
